# Isolation of Exosomes from Human Serum Using Gold-Nanoparticle-Coated Silicon Surface

**DOI:** 10.3390/nano13030387

**Published:** 2023-01-18

**Authors:** Krishna Thej Pammi Guru, Nusrat Praween, Palash Kumar Basu

**Affiliations:** Department of Avionics, Indian Institute of Space Science and Technology, Thiruvanathapuram 695547, India

**Keywords:** gold nanoparticle synthesis, exosome immobilization on the surface, isolation of exosomes, NTA, immunoaffinity technique

## Abstract

Exosomes, whose mean diameter ranges from 20 nm to 200 nm, are cell-secreted vesicles and are abundant in most biological fluids, such as blood, urine, tears, sweat, breast milk, etc. Exosomal size variations and their composition can be attributed to several factors, such as age, gender and disease conditions of the individual. Existing techniques, such as ultracentrifugation and density gradient ultracentrifugation, for exosome isolation are instrument-dependent, time-consuming and lack specificity. In the present work, a gold-nanoparticle (GNP)-coated silicon (Si) wafer, functionalized with polyethylene glycol (PEG) was used for conjugation with anti-CD63 antibody via EDC NHS chemistry and incubated with serum to immobilize the exosomes on the Si surface. The surface-immobilized exosomes were eluted and quantified by a nanoparticle tracking analyzer (NTA). It was observed that an increase in GNP density on the Si wafer increases the size range and total number of exosomes that are being isolated. Western blotting performed for proteins such as HSP 70 and calnexin confirmed the immobilization and elution of exosomes. The proposed technique can be used as an alternative to existing techniques, as it has several benefits such as reusability of the Si surface for several isolations, minimal instrumental requirement, isolation of exosomes in two hours and compatibility with the microfluidic platform, making the technique suitable for real-time application. The proposed method could be useful in isolating a specific subrange of exosomes by altering the size of the GNP used for coating the Si wafer.

## 1. Introduction

Exosomes are vesicles secreted from cells, which are considered to be crucial in intercellular communication [1] and are also involved in the secretion of proteins relevant to signaling pathways, which in turn induce exosome secretion [2]. Their mean diameter ranges from 20 nm to 200 nm and contains miRNA [3], DNA [4] and protein [5]. Exosomes exist in almost all body fluids, such as blood [6], urine [7], breast milk, saliva and plasma [8], and are found to be excess in cancer patients. Hence, they are considered crucial in the prognosis and diagnosis of cancer. Besides cancer, exosomes also play a significant role in several diseases related to Alzheimer’s, the brain, the immune system, the heart, etc. [9,10]. Though ultracentrifugation is one of the most standardized and extensively used techniques, several researchers are working toward finding better alternatives for isolating exosomes.

The two most widely used techniques for isolating exosomes that involve the usage of an ultracentrifuge are differential ultracentrifugation (UC) and density gradient ultracentrifugation (DGUC). Besides this, size-exclusion chromatography (SEC) is another frequently used technique. Apart from these techniques, there are several commercially available kits to isolate exosomes. In UC [11,12], the samples are spun at high speeds of 100,000× *g* to 200,000× *g*. Several variations exist in techniques involving UC, with respect to speed, time and volume of the starting solution being used. In DGUC [13], the sample solution is loaded in an inert gradient medium. After centrifugation, the exosomes settle down at their isodensity zone within the centrifuge tube. Besides exosomes, particles with a similar density to that of exosomes will also be coisolated [14] as contamination. SEC [15,16] is another popular method for isolating exosomes from biofluids. In SEC, the samples are centrifuged at a low speed in the initial stages to separate the cells, cell debris and other cellular organelles. The supernatant, containing exosomes, is passed through a porous filter paper, whose pore diameter (0.22 µm) is large enough to allow the particles in the exosomal size range to pass through. The enriched exosome solution is passed through an SEC column and fractions are collected at regular intervals to make the solution more enriched. The concentration of the isolated extracellular vesicles can be increased further using centrifugal filters. In SEC as well as in UC, plasma proteins such as albumin are coisolated along with the extracellular vesicles [17], as contamination is undesirable and unavoidable. Hence, immunoaffinity techniques are preferred to enhance the specificity in exosome isolation techniques.

One such technique involving immunoaffinity-based exosome isolation employs the usage of magnetic beads [18]. A solution devoid of cell and cell debris was incubated with magnetic beads functionalized with anti-CD81 antibody at 4 °C for 16 h. After incubation, the solution containing the beads/exosome complex was pulled to one side of the centrifuge tube using a magnet, followed by replacing the supernatant with PBS. Besides these techniques, there are several commercial kits available that can isolate exosomes. Such kits are miRCURY, ExoQuick and TEIR, which can isolate approximately 10^9^ particles from 250 µL of serum, whose mean diameter is 120 ± 3 nm. Contaminants such as cell debris, aggregated proteins and lipoproteins are to be expected in techniques that employ the precipitation method for isolating exosomes [19]. The advantages and disadvantages of all the well-known techniques are presented in one of our earlier works [20].

Another group isolated extracellular vesicles (EVs) from serum using a monolithic column [21]. The column, containing a methacrylate monolithic disc, was functionalized with anti-CD61 antibody five times a day for three days. After functionalization, a syringe pump was used to flow the serum through the column, to immobilize the EVs on the disk. The immobilized EVs in the column were eluted using carbonate/bicarbonate solution at pH 11.3. Though this method is unique, the processing steps are time-consuming. Another method to isolate EVs is by using Tim4 protein, as it specifically binds to phosphatidylserine, which is expressed in all EVs. EVs from the K562 cell line were isolated by incubating the cell-free medium with Tim4-functionalized magnetic beads in the Ca^2+^ environment [22]. The exosomes bound to the beads can be eluted by using EDTA as a chelating agent.

Our group reported another immunoaffinity method for isolating exosomes from human serum by using a bench-top centrifuge [20]. Gold nanoparticles (GNPs) functionalized with anti-CD63 antibody were used for conjugation with exosomes in human serum for 1 h. Due to excess weight on the exosomes, caused by the conjugation with GNPs, they settled down and formed a pellet at a very low g-force.

In the present work, we report a GNP-based, quick and simple immunoaffinity technique for the isolation of exosomes. GNPs coated on a Si wafer were conjugated to polyethylene glycol (PEG), whose one end was a carboxyl group (COOH), and exosomal surface protein CD63 was conjugated to the COOH on the Si surface by ethyl (dimethyl aminopropyl) carbodiimide (EDC) and hydroxy succinimide (NHS) chemistry. The antibody-functionalized Si wafer was incubated with serum to immobilize the exosomes on the Si surface. The exosomes were immobilized on the surface in less than two hours and were confirmed by techniques, such as using a nanoparticle tracking analyzer (NTA) and Western blotting. It was observed that the size of the GNPs may play a significant role in isolating different sizes of exosomes.

## 2. Materials and Methods

### 2.1. Materials

Gold (III) chloride hydrate (HAuCl4—Cat No. 254169), trisodium citrate dihydrate (Cat No. S1804), poly(ethylene glycol) 2-mercaptoethanol ether acetic acid (PEG 3500 Da—Cat No. 757837), 2-(N-morpholino) ethanesulfonic acid (MES—Cat No. M5287), N-hydroxysuccinimide (NHS—Cat No. 56480), N-(3-dimethylaminopropyl)-N-ethyl carbodiimide (EDC—Cat No. 39391), and bovine serum albumin (Cat No. 05470) were procured from Sigma-Aldrich (St. Louis, MO, USA). Nonfat milk powder and clot activator tubes (4 mL) were procured from local vendors. Amersham ECL reagent (Cat No. RPN2235) was obtained from GE HealthCare (Chicago, IL, USA). All other reagents and consumables were of reagent grade. Primary antibodies anti-HSP70 (Cat No. MAb 33-3800), anti-CD63 (Cat No. BS-1523R) and anti-calnexin (Cat No. MA5-15389) were procured from Thermo Fisher (Waltham, MA, USA). Secondary antibodies anti-mouse (Cat No. ab131368) HRP and anti-rabbit (Cat No. ab131366) HRP were obtained from Abcam (Cambridge, UK).

### 2.2. Methods

**Figure 1:**
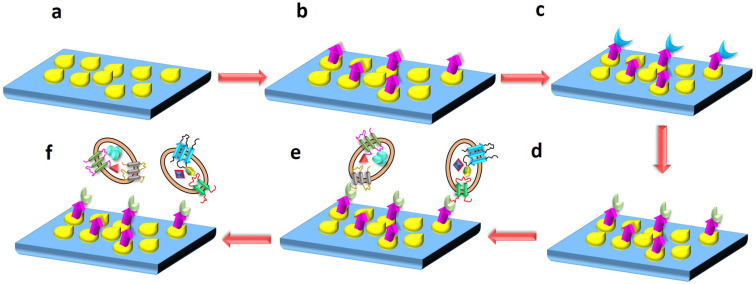
Procedure to immobilize and elute exosomes from Si surface coated with gold nanoparticles (GNPs)**:** (**a**) Si wafer coated with GNPs; (**b**) conjugation of polyethylene glycol (PEG) to the GNPs; (**c**) EDC NHS activation of PEG; (**d**) PEG on the Si surface conjugated with anti-CD63 antibody; (**e**) exosome immobilization on the Si surface after serum incubation; (**f**) elution of exosomes from the Si surface.

In the present work, exosomes were immobilized on the GNP-coated silicon (Si) wafer by the immunoaffinity technique. PEG was immobilized on the Si wafer, which was coated with GNPs. The carboxyl group (COOH) of the PEG was activated with EDC NHS chemistry and functionalized with anti-CD63 antibody. After functionalization, serum was incubated on the Si wafer to immobilize the exosomes on it. Figure 1 depicts the stages involved in the immobilization of exosomes on the Si surface.

#### 2.2.1. GNP Preparation

Stock solutions of gold (III) chloride trihydrate and trisodium citrate dihydrate (TCD) were prepared at molarities of 4.2 mM and 17 mM, respectively. A 1 mL volume of gold (III) chloride trihydrate solution was mixed with 18 mL of DI water and heated in a beaker at 80 °C. A 1 mL volume of TCD was added to the boiling gold solution and left for heating till the solution turned amber red, after which the solution was cooled down to room temperature. After the gold solution reached room temperature, it was stored at 4 °C for further usage.

#### 2.2.2. Si Wafer Coating with GNPs

The Si wafer diced in 1 cm × 1 cm dimension was washed with DI water on a rocker to remove grains of dust. After washing, the Si wafer was heated on a hot plate at 300 °C. While heating the Si wafer, GNP solution was drop-casted on the Si wafer at a flow rate of 50 µL/min using a syringe pump. Different Si wafers were prepared by dispensing different volumes of GNP solution (4 mL, 8 mL, 12 mL). After the deposition of the GNP solution, the Si wafers were annealed for 4 h at 300 °C and cooled down to room temperature before isolating exosomes.

#### 2.2.3. PEGylation of Si Wafer and Activation of COOH of the PEG via EDC NHS Chemistry

PEG solutions of different concentrations (28.5 µM, 285 µM, 857 µM, 1.42 mM) were prepared in DI water, and 200 µL was used for incubation on the Si wafer for 2 h at room temperature. After incubation, the Si surface was washed with DI water to remove unconjugated PEG from the Si surface. 

The carboxyl group (COOH) of the PEG was activated using EDC NHS coupling chemistry. In 200 µL of MES buffer (pH 6.17, 0.1 M), 20 µL of 10% NHS and EDC were mixed and incubated on the PEGylated Si wafer for 30 min at room temperature. Different experiments were performed by varying the concentrations of EDC (14 mM, 28 mM, 56 mM, 141 mM, 282 mM).

#### 2.2.4. Functionalizing the Si Surface with Anti-CD63 Antibody and Immobilization of Exosomes

After activating the COOH of the PEG, the Si surface was incubated with 200 µL of sodium phosphate buffer (SPB) containing 1 µL of anti-CD63 antibody for 30 min at 4 °C, followed by washing of the Si wafer with washing buffer for 10 min at 4 °C to remove unbound antibody. The serum (100 µL) diluted with 100 µL of SPB was incubated on the Si wafer for different durations (10 min, 20 min and 30 min) at 4 °C, followed by washing the Si wafer with washing solution (for 10 min at 4 °C).

#### 2.2.5. Elution of Exosomes from the Si Surface and NTA Quantification

To quantify the number of exosomes immobilized on the Si wafer, the Si surface functionalized with 1 µL of anti-CD63 antibody was incubated with 200 µL of diluted serum solution (100 µL of serum and 100 µL of SPB) for 30 min at 4 °C. After incubation, the Si wafer was washed with washing solution for 10 min at 4 °C. To elute the exosomes immobilized on the surface, the Si wafer was incubated with 40 µL of glycine buffer (0.1 M, pH-3) for 50 min at room temperature, followed by addition of 1 M NaOH to neutralize the pH of the solution. The exosomal solution was mixed with an equal volume of modified Karnovsky fixative solution and used for NTA analysis using NanoSight NS300 (Malvern Panalytical Instruments, Malvern, UK).

#### 2.2.6. Serum Preparation

Blood collected from a healthy volunteer was used for preparing serum. The RBCs of blood were clotted in clot activator tubes for 1 h at room temperature in an upright position. The tube containing blood was spun at 7000× *g* at ambient temperature for 20 min. The serum, which was the supernatant, was collected using a pipette. Aliquots of 100 µL of serum were prepared in 1.5 mL centrifugation tubes and stored at −20 °C for further experiments. For each experiment, 100 µL of serum was diluted with 100 µL of SPB before performing the experiment.

#### 2.2.7. Western Blotting

After immobilization of exosomes on the Si surface, the exosomes were eluted with 50 µL of 0.1 M glycine buffer at pH 3 for 50 min at room temperature, followed by addition of 1 M NaOH to neutralize the pH of the solution. The solution containing exosomes was treated with SDS sample loading buffer and heated for 10 min at 97 °C. These samples were run in 10% SDS-PAGE, followed by blotting in the PVDF membrane. The blot was blocked with 5% nonfat milk for 1 h at room temperature. The PVDF membrane was probed with suitable primary antibodies at 4 °C overnight, followed by conjugation with HRP-conjugated secondary antibody. The blots were developed by using ECL reagents. 

#### 2.2.8. SEM Characterization of Si Wafer Coated with GNPs

Si wafers drop-casted with different volumes of GNP solution (4 mL, 8 mL, 12 mL) were annealed at 300 °C for 4 h. After annealing, the samples were used for surface characterization using SEM.

#### 2.2.9. Optimization of Washing Solution

A Si wafer coated with GNP solution was incubated at room temperature for 30 min with various concentrations of HRP-tagged secondary antibody (2 µL, 5 µL, 10 µL). After incubation, the Si wafer was washed with solutions such as NaCl (0.5 M and 1 M), sodium phosphate buffer (SPB) (0.25 mM, 0.1 M, 0.5 M, and 1 M), Triton X-100 (0.1%, 1% and 10%), 0.1% Tween 20, and a solution containing 0.1% Tween 20 and 5% Triton X-100. The washing steps were performed for varying amounts of time, ranging from 15 to 45 min, and one to three times. On the Si wafer, TMB was incubated following washing. 

PEGylated Si wafer was incubated for 30 min at room temperature with 1 µL of secondary antibody labeled with HRP. After incubation, the Si wafer was washed for 10 min with Tween 20 (0.1%), Triton X-100 (0.1%), 0.1 M SPB and 0.5 M NaCl, and then it was incubated with TMB.

## 3. Results and Discussions

### 3.1. Preparation of GNPs and Coating Si Wafer

**Figure 2:**
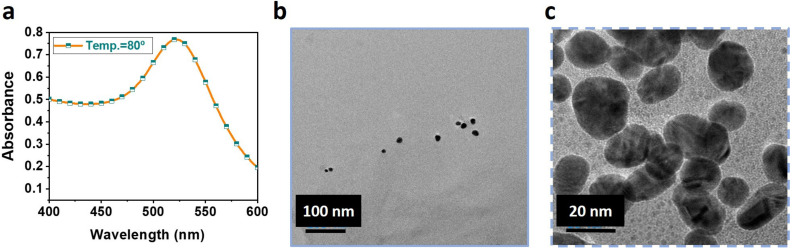
Synthesis of gold nanoparticles (GNPs): (**a**) UV–VIS characterization of GNPs; transmission electron microscope (TEM) characterization of GNPs at (**b**) 100 nm resolution and (**c**) 20 nm resolution.

GNPs were synthesized by the Turkevich method [23] using gold (III) chloride trihydrate and TCD. The synthesis depends on several factors, such as temperature, concentrations of gold (III) chloride and TCD. Several experiments were performed by varying all possible parameters to synthesize stable GNPs in our earlier [20] works. In brief, GNPs were prepared at 80 °C, with 1 mL each of gold (III) chloride trihydrate and TCD stock solutions of molarities 4.2 mM and 17 mM, respectively, in 18 mL of DI water. Figure 2a shows the UV–VIS characterization of GNPs, which can be used to determine the concentration of GNPs [24]. The concentration was found to be 7.4 × 10^8^ per mL. Figure 2b,c shows the TEM images of the GNPs at resolutions of 100 nm and 20 nm, respectively. The results show that the majority of the synthesized particles are of mean diameter 20 nm.

The Si wafer was diced into 1 cm × 1 cm dimensions using a diamond cutter and washed in a Petri dish with DI water till the grains of dust were completely removed. After washing the Si wafer, it was dried on a hot plate by heating at 300 °C, and the temperature was maintained for dispensing GNPs on the Si wafer. A syringe pump was used to drop-cast the GNP solution at a flow rate of 50 µL per min. Different samples were prepared by depositing different volumes of GNP solution (12 mL, 8 mL, 4 mL) on the Si wafer, which was annealed for 4 h at 300 °C. Finally, the wafer was cooled down to room temperature before using it for further processing steps.

### 3.2. Optimizing the Washing Solution for Elimination of Nonspecific Adsorption

**Figure 3:**
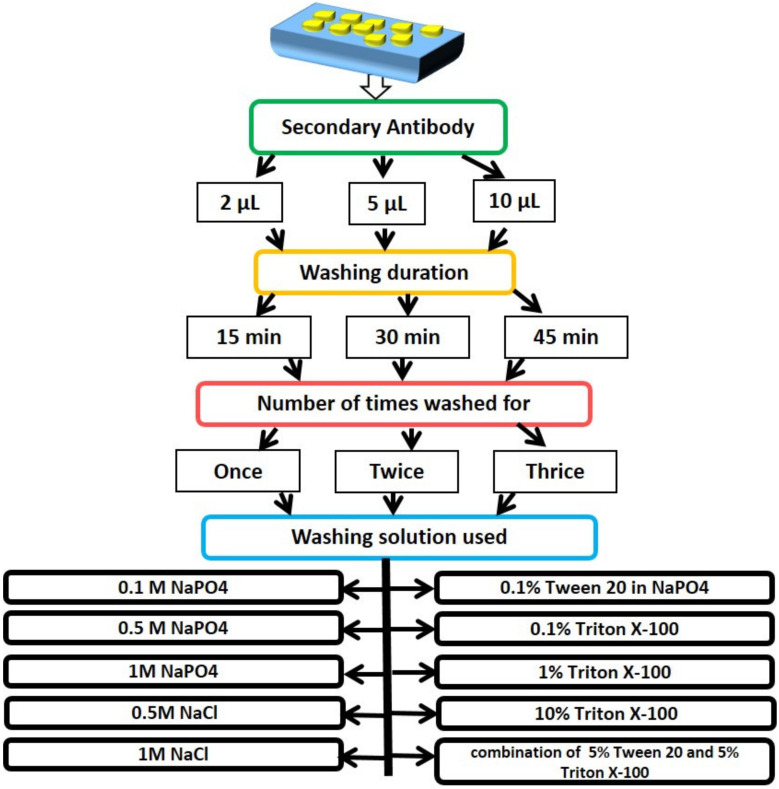
Experiments performed to optimize a washing solution to eliminate nonspecific adsorption. Different concentrations of secondary antibody were incubated on the gold-nanoparticle-coated Si wafer, washed for different durations (multiple times) with different types of detergents and salt solutions (of different molarities).

Elimination of nonspecific adsorption is very crucial in immunoaffinity techniques, as the surface is exposed to biomolecules several times during the incubation process. To test the efficiency of different washing solutions to wash off the adsorbed antibody on the GNP surface, various salt and detergent solutions were prepared and used. Proteins, which are both hydrophilic and hydrophobic in nature, get adsorbed to the GNP surface through hydrogen bond, sulfur-containing amino acids, hydrophobic interactions, etc. In order to understand the adsorption strength of proteins on the gold surface, secondary antibody tagged with HRP was incubated on the GNP-coated Si surface for 30 min at room temperature and washed with various solutions: NaCl (0.5 M and 1 M), sodium phosphate buffer (SPB) (0.25 mM, 0.1 M, 0.5 M and 1 M), Triton X-100 (0.1%, 1% and 10%), 0.1% Tween 20, and solution with a combination of 5% Tween 20 and 5% Triton X-100. After washing, the Si surface was introduced to TMB solution. A significant color change was noticed after 2 min in TMB solution, i.e., the TMB turned blue from colorless, indicating the presence of adsorbed secondary antibody on the Si surface even after washing. Several experiments that were performed are detailed in Figure 3. It was concluded that removal of the adsorbed proteins from the gold surface, even with highly concentrated salts or detergents, is a difficult task, and hence, in order to avoid the nonspecific adsorption, PEG was immobilized on the surface of the SPE.

### 3.3. PEGylation of Si Surface

Different concentrations of PEG solutions (28.5 µM, 285 µM, 857 µM, 1.42 mM) were prepared in DI water. A 200 µL volume of PEG solution was incubated with the GNP-coated Si surface for 2 h and washed with 25 mM SPB. Following washing, the Si wafer was incubated with SPB mixed with 1 µL of secondary antibody for 30 min, and after that, the Si surface was washed for 10 min with 0.1% Tween 20 solution and introduced to TMB. It was observed that the rate of change in the color of TMB was relatively less (but not negligible) as compared to that of the Si surface without PEG. Experiments were also performed by changing the washing solutions (Triton X-100, 0.1 M SPB, 0.5 M NaCl), and it was noticed that the PEG concentrations of 285 µM, 857 µM and 1.42 mM gave comparable visible results. It was concluded from the performed experiments that 0.1% Tween 20 and 0.1% Triton X-100 are efficient in the removal of the adsorbed secondary antibody compared to that of 0.5 M NaCl and 0.1 M SPB. Since Tween 20 is a milder detergent, it was used as washing solution for further experiments. 

### 3.4. Activation of COOH of PEG by EDC NHS Coupling

EDC NHS is a well-known coupling chemistry used for establishing a link between COOH groups and the antibody, and it is optimum at acidic pH. Hence, MES buffer was chosen for this purpose. Our research group earlier optimized the pH of MES buffer to 6.17. In 200 µL of 0.1 M MES buffer of pH 6.17, 20 µL of 10% NHS and different concentrations of EDC (14 mM, 28 mM, 56 mM, 141 mM, 282 mM) were mixed and incubated for 30 min at room temperature, followed by incubation with 200 µL of SPB containing 1 µL of secondary antibody for 30 min at 4 °C. Next, the Si wafer was washed with washing buffer for 10 min at 4 °C to remove unbound antibodies from the Si surface, and finally, it was incubated with TMB for 2 min. The sample prepared with 28 mM of EDC showed a significant change in color of TMB, indicating the presence of more secondary antibody on the Si surface. It is inferred that lower concentrations of EDC might not have activated a significant number of COOH groups of the PEG, and hence there might be a smaller number of binding sites for the antibody to bind. Higher concentrations of EDC increase the pH of the MES solution, and the activation of COOH might not be optimum. Hence, an EDC concentration of 28 mM was used for all further experiments.

### 3.5. Functionalization of Si surface with Anti-CD63 Antibody and Immobilization of Exosomes

The COOH-activated PEG was incubated with 200 µL of SPB containing 1 µL of CD63 antibody for 30 min at 4 °C. Next, the Si wafer was washed with washing solution for 10 min at 4 °C and was incubated with 200 µL of diluted human serum (100 µL serum with 100 µL SPB) for 30 min. Following serum incubation, the exosome samples were eluted and used for electrophoresis and Western blotting. The Si wafer was also incubated with serum for 10 min and 20 min, and the corresponding samples were also used for Western blotting. Since there was no visible band for the samples prepared with 10 min and 20 min incubation time, we assumed that a minimum of 30 min incubation is needed for the conjugation to happen between the antibody and the exosome.

### 3.6. NTA Characterization of Exosomes

**Figure 4:**
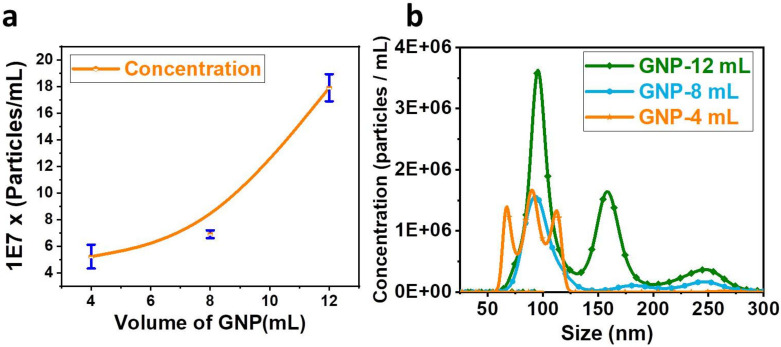
Characterization of exosomes by nanoparticle tracking analyzer (NTA): (**a**) Concentration of the particles with respect to the volume of the gold nanoparticles (GNPs) used for deposition on the Si wafer. Figure shows mean and Standard Error of Mean of three sets of data. The graph shows significant increase in the number of isolated exosomes with the increase in the Volume of GNP used for coating the Si wafer (**b**) concentration of the particles isolated from Si wafers coated with different volumes of the GNP solution. Figure shows the isolation of smaller particles from the Si wafer deposited with less volume of GNP compared to Si wafer deposited with higher volume of GNP solution.

In order to conduct NTA characterization, a Si wafer that had been coated with varying volumes of GNPs (12 mL, 8 mL, and 4 mL) was incubated with serum and then eluted. The total number of exosomes isolated significantly increased from 5 × 10^7^ particles/mL to 17 × 10^7^ particles/mL when the volume of GNP deposition was increased from 4 mL to 12 mL, as shown in Figure 4a. This change was brought about by an increase from 4 mL to 12 mL. The numerical evidence demonstrates that an increase in the total number of particles isolated from serum is accompanied by an increase in the number of GNPs deposited on the surface of the Si. Increasing the amount of GNP solution that is applied to the Si wafer during the coating process results in an increase in the surface area of the Si wafer that is available for the conjugation of PEG. This, in turn, results in an increase in the amount of antibody conjugation, which maximizes the exosome isolation to 17 × 10^7^ particles/mL. 

The inference is supported by the findings shown in Figure 4b. Si wafers coated with 12 mL of GNPs were used to isolate 17 × 10^7^ particles/mL, covering the exosome diameter range of 70 to 200 nm. A significant number of particles smaller than 100 nm were separated using a Si wafer that had been coated with 4 mL of GNP solution. On the other hand, a Si wafer that had been coated with 8 mL of GNP had isolated particles with a diameter that is somewhere in the middle of that of the other two volumes (4 mL, 12 mL). Based on the findings of the NTA, we have reason to believe that it is possible to achieve selective isolation of exosomes by adjusting the size of the nanoparticle deposited on the Si wafer.

### 3.7. SEM Characterization of Si Wafer Coated with GNPs

**Figure 5:**
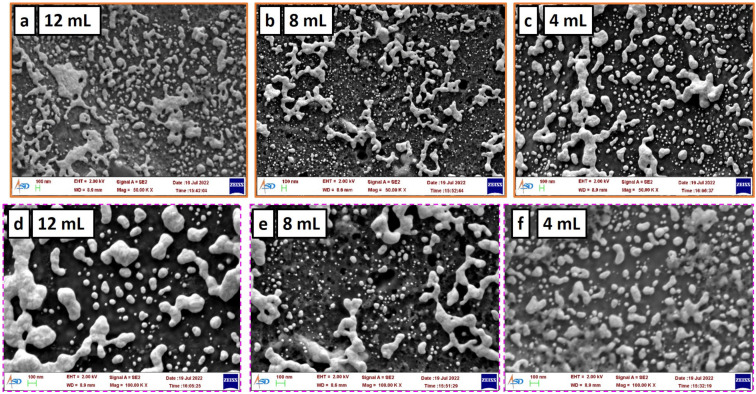
Scanning electron microscope (SEM): characterization of Si surface deposited with different volumes of GNP solution: 50× resolution: (**a**) 12 mL; (**b**) 8 mL; (**c**) 4 mL; 100× resolution: (**d**) 12 mL; (**e**) 8 mL; (**f**) 4 mL; Figure shows the formation of different-sized gold nanoparticle (GNP) aggregates on the Si wafer deposited with different volumes of GNP solution.

Si wafers that had been coated with 12 mL, 8 mL and 4 mL of GNPs were utilized for the SEM characterization process. Figure 5 illustrates the scanning electron microscopy (SEM) analysis of the wafers at two different resolutions, namely, 100× and 50×. The gold coverage on the surface of the Si wafer coated with 12 mL of GNP solution is more continuous and uniform when contrasted with the gold coverage on the surface of the Si wafer coated with 4 mL of GNP solution (Figure 5). It is possible that the PEG density is highest in the part of the Si wafer where there are more GNPs in close proximity to one another (Figure 5a,d). If there are a greater number of PEG molecules present on the surface of the silicon wafer, then there is a greater possibility that antibodies will become attached to the PEG that is located there. The presence of a greater number of antibodies in a specific region may generate an environment that is more amenable to the isolation of larger exosomes than would otherwise be the case. 

The SEM image of a Si wafer that has been coated with 8 mL of GNP solution can be seen in Figure 5b,e. The upper range of the gold clusters on wafers coated with an 8 mL GNP solution is lower compared to that of wafers coated with a 12 mL GNP solution. The minimum size of the gold cluster is smaller than the gold clusters that were formed on the Si wafer that had been coated with 12 mL and 4 mL of GNP solution. The process of coating the Si wafer with 8 mL can be thought of as a staging area between the application of 12 and 4 mL volumes. The results of the NTA shown in Figure 4b show that the sample deposited with 8 mL of GNP solution was able to successfully isolate exosomes of both smaller and larger diameters. Figure 5c,f, shows the Si wafer coated with 4 mL of GNP solution, which shows that the majority of the gold aggregates are of uniform size and they are not too small and too big. It can be inferred that the too-small surface area formed by depositing GNPs may not be efficient in capturing smaller-diameter exosomes. 

It is possible to draw the following conclusion based on the data presented in Figure 4 and Figure 5: the surface area availability of GNPs, as well as the gap between the nanoparticle aggregates, plays an important role in the process of isolating exosomes of a specific diameter. The surface of the Si wafer is made to be clean during the manufacturing process by undergoing multiple rounds of polishing. Because the surface roughness cannot be controlled accurately during the polishing steps, there will be pores on the Si surface that are not uniform in size. Consequently, the pores will not be uniformly distributed. This can result in the GNP clusters on the Si surface being distributed in an uneven manner. However, if the pore diameter of the surface can be controlled, then it will be possible to achieve the uniformity of GNP clusters on the surface, which will be helpful in selectively isolating particles. 

**Figure 6:**
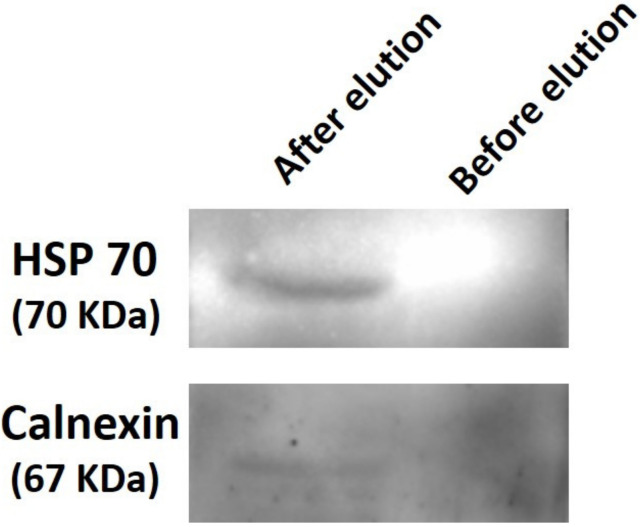
Western blot performed with exosomes eluted from the Si surface. The blot shows the Western blot results of SDS-PAGE loaded with samples obtained from the Si surface, before and after elution. A 50 µL volume of solution was used for the experiment. The blot was incubated with primary antibody overnight and secondary antibody for 1 h at room temperature.

For the purposes of electrophoresis and Western blotting, exosomes that had been immobilized on a Si wafer by coating with 12 mL of GNP solution were used. In order to accomplish this, the immobilized exosomes were eluted from the Si surface using 50 µL of 0.1 M glycine buffer at a pH of 3 and then subjected to sample loading buffer. After immobilizing exosomes on a Si wafer and allowing it to incubate with SPB at room temperature, the SPB was then used as a reference sample. Figure 6 displays the representation of the blot probed with HSP 70 and calnexin. The presence of dark and light bands for HSP70 and calnexin, respectively, demonstrates that the corresponding proteins were either over- or underexpressed. The results of the blot are consistent with the existing research [25], which states that calnexin should not be present in exosomes or should have very low levels of expression. The presence of a light band for calnexin demonstrates that the majority of the particles that were isolated on the Si wafer were exosomes. This is demonstrated by the presence of the light band. 

## 4. Conclusions

Gold nanoparticles of diameter 20 nm were synthesized and used for coating a Si wafer by drop-casting using a hot plate. These Si wafers were PEGylated and functionalized with anti-CD63 antibody to specifically isolate exosomes from serum. The isolated exosomes were eluted and quantified using NTA. The eluted exosomes were also used for Western blotting to determine the presence of exosomal and nonexosomal proteins. Increasing the volume of GNP deposition on the Si wafer from 4 mL to 12 mL increased the total exosome isolation by 240% (i.e., from 5 × 10^7^ to 17 × 10^7^). Further, the Si wafer deposited with a higher volume of GNPs (12 mL) could isolate bigger exosomes (70 nm to 200 nm) compared to the Si wafer prepared with a low volume (4 mL) GNP (60 nm to 125 nm). The isolation of exosomes could be achieved in less than 2 h. From the results, it can also be concluded that altering the size and deposition volume of GNPs on the Si wafer could be useful in isolating a specific subrange of exosomes. The technique has benefits, such as reusability of the Si wafer for several isolations, and involves a simple and minimum number of instruments, making it easily repeatable by researchers for exosomal studies. The proposed technique can also be integrated with microfluidic systems.

## Data Availability

Not applicable.
